# Influence of Electrolyte Composition on the Semiconductor–Electrolyte Interface (SEI) Built-In for Enhanced Photoelectrochemical (PEC) Processes

**DOI:** 10.3390/molecules30040885

**Published:** 2025-02-14

**Authors:** Bartłomiej Leks, Aleksandra Parzuch, Nabila Nawaz, Justyna Widera-Kalinowska, Krzysztof Bienkowski, Renata Solarska

**Affiliations:** 1 Laboratory of Molecular Research for Solar Energy Innovations, Centre of New Technologies, University of Warsaw, Banacha 2c, 02-097 Warsaw, Poland; b.leks@cent.uw.edu.pl (B.L.); a.parzuch@cent.uw.edu.pl (A.P.); n.nawaz@cent.uw.edu.pl (N.N.); 2 Department of Chemistry, Adelphi University, 1 South Avenue, Garden City, NY 11530, USA; widera@adelphi.edu

**Keywords:** cuprous oxide Cu_2_O, solar energy conversion, photocatalyst, electrochemical double layer, carbonates

## Abstract

The relentless consumption of fossil fuels and soaring CO_2_ emissions have plunged the world into an energy and environmental crisis. As society grapples with these challenges, the demand for clean, renewable, and sustainable energy solutions has never been more urgent. However, even though many efforts have been made in this field, there is still room for improvement concerning efficiency, material stability, and catalytic enhancement regarding kinetics and selectivity of photoelectrochemical (PEC) processes. Herein, we provide the experimental proof for the enhancement of the photocurrent efficiency by the critical focus on semiconductor–electrolyte interface (SEI) properties. By tailoring electrolyte composition, researchers can unlock significant improvements in catalytic efficiency and stability, paving the way for advanced PEC technologies. In this study, we investigate the influence of electrolyte composition on SEI properties and its impact on PEC performance. By employing electrolytes enriched with carbonates, borates, sulphates, and alkali cations, we demonstrate their profound role in optimising photoelectrochemical CO_2_ reduction reaction (CO_2_RR) efficiency. Central to this work is Cu_2_O—an affordable, highly promising photocatalyst. While its potential is undeniable, Cu_2_O’s inherent instability and diverse reduction products, ranging from CH_3_OH to CO, HCOOH, CH_3_COOH, and CH_3_CH_2_OH, have hindered its widespread adoption in PEC CO_2_ reduction (CO_2_RR). Our approach leverages a straightforward yet powerful electrodeposition method, enabling a deeper exploration of SEI dynamics during photocatalysis. Key parameters, such as carbonate concentration, local pH, alkali cation presence, anionic geometry, CO_2_ solubility, and electrolyte conductivity, are systematically investigated. The findings reveal the formation of a unique “rigid layer” at the photocatalyst surface, driven by specific cation–anion interactions. This rigid layer plays a pivotal role in boosting PEC performance, offering a new perspective on optimising, among other PEC processes, CO_2_RR catalytic efficiency. This profound study bridges a critical knowledge gap, shedding light on the dual influence of cations and anions on SEI properties and PEC CO_2_RR. By unravelling these intricate interactions, we provide a roadmap for designing next-generation PEC systems. These insights pave the way for sustainable energy advancements, inspiring innovative strategies to tackle one of the most pressing challenges of our time.

## 1. Introduction

The application of a huge number of photoactive materials has already been described in the literature for CO_2_ photoreduction; however, most of them present many disadvantages of instability, poor selectivity, low energy conversion efficiency, photocorrosion, and inability to entirely suppress the competitive hydrogen evolution reaction (HER) that occurs in the presence of water [1,2,3,4]. These problems remain unresolved to this day. Moreover, in aqueous electrolytes, water plays several roles. It works as a solvent, a sacrificial reducing agent, and an abundant source of protons crucial for the proton-coupled electron transfer (PCET) in the PEC reduction of CO_2_. The low solubility of CO_2_ in water (∼35 mM at 1 atm) [5] restricts the diffusion of CO_2_ to the photocathode and inhibits the process of CO_2_ reduction. In this regard, electrolyte is also a key factor in PEC CO_2_ reduction and its transformation into solar fuels. Ionic species dissolved in a proper solvent form an electrolyte that acts as an ionic conductor, enabling charge compensation on each electrode in the PEC cell [6]. A Schottky junction will be formed at the electrode/electrolyte interface upon the immersion of an electrode in an electrolyte. Due to this fact, the build-up of the electric field in the depletion zone induces the upward bending of the conduction band (CB) and valence band (VB) observed at the electrode/electrolyte interface [7]. The essential aspect of achieving high product selectivity and high Faradaic conversion efficiency is the proper composition of a solution serving as an electrolyte for the PEC CO_2_ reduction. Zanoni and co-workers [8] obtained different products on the copper electrodes depending on the applied pH. For instance, ethanol, methanol, acetaldehyde, and formaldehyde were generated at pH 9; moreover, higher selectivity but lower conversion efficiency were achieved at higher pH values. It must be noted that the composition of the electrolyte will vary with the pH of the solution. Recently, various reports have been published demonstrating the application of various solutions serving as electrolytes for CO_2_ conversion into solar fuels via PEC reactions. Overall, they can be categorised as aqueous or non-aqueous solutions containing either the electron donors or the sacrificial reducing agents.

Effective conversion of CO_2_ to methanol by using a cuprous oxide as a photocathode was first demonstrated by Rajeshwar [9]. His group demonstrated the detection of methanol formed from the PEC CO_2_ reduction on hybrid CuO/Cu_2_O nanorod assemblies. In the recent literature, there are many papers that demonstrate the production of different products on Cu_2_O [10,11,12]. However, it is important to note that selectivity is crucial. Therefore, the electrolyte composition, its nature, and concentration have an obvious and vital impact on the CO_2_RR pathway by influencing the reaction environment adjacent to the electrode [13,14,15,16]. In addition, the electrolyte may influence also the structure of Cu_2_O, due to the direct contact between them. However, the PEC CO_2_RR mechanism is not well understood in terms of the influence effecting from the changes in Cu_2_O structure and electrolyte composition on the product distribution. In the present work, we try to elucidate the role of the electrolyte and the influence of the alkali cations on the solar to fuel efficiency and CO_2_RR product distribution. The pH value of the applied electrolyte depends on the type of cation and anion species and their respective concentrations. The presence of both, cations and anions impact the CO_2_RR in diverse ways. Already in the 1970s, Paik et al. showed the first electrocatalytic CO_2_RR to be dependent on the presence of the alkali cations of: Li^+^, K^+^, Na^+^, Cs^+^, and NH_4_^+^. However, the main reaction products were formic acid and H_2_ (through the HER) [13]. Twenty years later, Murata, Hori et al. showed increased FEs promoted by larger cation sizes (Li^+^ < Na^+^ < K^+^ < Cs^+^) [17]. The size effect was attributed to the reduced overpotentials for larger electrolyte cations, which led to better adsorption of a given cation at the cathode, causing an effective potential difference between the cathode and the electrolyte. The occurring potential difference favoured a reduction in neutral species (CO_2_) over the positively charged H^+^, hence suppressing the HER in an acidic medium [18]. The product distribution of CO_2_RR carried out at the Cu electrode and in bicarbonate solutions towards ethylene and alcohols was investigated by Kyriacou et al. [19]. They found out that the cation influence on CO_2_RR could be enhanced by the increase in the CO_2_ partial pressure. Both current efficiency and the rates of formation of the reduction products were linearly dependent on the CO_2_ pressure: both parameters diminished with decreasing CO_2_ pressure, while the evolution of hydrogen increased. Similarly to the previous work of Hori et al., the CO_2_RR product distribution towards C_2_H_4_ increased with the cation radius as follows: Li^+^ < Na^+^ < K^+^~Cs^+^. The non-metallic cation of NH_4_^+^, allowed, however, for the evolution of hydrogen with a CE of 92%. In their subsequent work, they confirmed that CO_2_RR carried out in the NH_2_HCO_3_ electrolyte led nearly exclusively towards the HER [13]. Despite the fact that the alkali cation influence on CO_2_RR could not be neglected, a satisfactory explanation for this phenomenon has not been developed and several mechanisms have been proposed, such as: (i) the favourable adsorption of large cations on the electrode, which leads to the stabilisation of CO_2_RR intermediates [20,21]; (ii) direct evidence of local pH change and the change in the local pH and buffering ability for large cations occurring due to the low pK_a_ for cation hydrolysis at the vicinity of the electrode [22,23]; and (iii) differences in overpotentials caused by the electrochemical potential in the outer Helmholtz plane affected by different cations [18,20]. The latter mechanism has been developed further on and supported by a DFT calculation by J. Resasco et al. [24]. The authors carried out the experimental work under conditions, where the influence of the electrolyte polarisation was minimal in order to elucidate the influence of each cation size on the intrinsic rates of formation of: HCOO^−^, C_2_H_4_, and C_2_H_5_OH over Cu(100), Cu(111)-oriented films, and over polycrystalline Ag and Sn. This experimental approach, supported by DFT results, led them to conclude that alkali cations influence the distribution of CO_2_RR products because of electrostatic interactions between solvated cations present in the outer Helmholtz plane and adsorbed species with large dipole moments.

A discussion of the influence and impact of CO_2_RR was provided by Sing et al. [25]. The main conclusion of their study is that the best pH is close to neutral; the influence of an additional electrolyte increases conductivity but decreases polarisation (reducing the electric field near the electrode and solubility of CO_2_; the addition of a pH buffer may affect selectivity). They also find that potential losses can be reduced more than anions rather than cations. Indeed, the interfacial properties at the electrode/electrolyte boundary have an important effect on the surface reactivity and, subsequently, on the catalytic efficiency of the CO_2_RR. To illustrate such importance, ionic additives have been added to the electrolyte to serve as promoters for specific reaction pathways. The authors claimed that the presence of positively charged trimethylammonium surfactant molecules effectively displaced the alkali cations and suppressed the generation of hydrogen. Thus, a molecular model of a double layer, assuming the CTAB molecule as an agent competing for a position at the outer Helmholtz plane, which results in only a small concentration of electrolyte cation at the electrode surface, has been proposed [26]. What is more, in view of the complex nature of the processes occurring in the double layer formed at the electrode/electrolyte interface, the role of anions in the presence of alkali cations cannot be neglected. Indeed, K. Ogura et al. reported an improvement in electrochemical CO_2_ reduction through specifically adsorbed halide anions to the vacant orbital of CO_2_. Those anions were claimed to suppress the adsorption of protons and led to a higher hydrogen overpotential, thus favouring the rate of electrochemical CO_2_ reduction [27]. This improvement was due to electron transfer from the electrode to CO_2_, modulated by halide anions. On the other hand, J. Resasco et al. observed the correlation between the effectiveness of CO_2_RR and the ability of buffering anions to donate hydrogen directly to the electrode surface [28]. Recently, such investigations have been performed for a photocatalytic CO_2_ reduction with the use of a semiconductor/complex CdS/Co(bpy)_5_Cl_2_) system. The authors investigated the influence of not only the halide anions but also of sulphates or phosphates [29]. They concluded that in their system, the anions such as NO_3_^−^, H_2_PO_4_^−^, and SO_4_^2−^ negatively affected CO_2_ reduction, while CO_3_^2−^ promoted the catalytic process of CO_2_ only when added in a modest amount. From this perspective, and keeping in mind often contradictory results, in this work, we present a new understanding of cations’ and anions’ role in the enhancement of CO_2_RR and the investigations of the impact of the local reaction environment around the catalyst. Understanding the interplay between the cation–anion interaction in the electrolyte and subsequently with the electrode surface will provide significant information about the composition of a double layer facilitating the electron transfer to CO_2_.

## 2. Results and Discussion

### 2.1. Morphology and Structure

**Figure 1:**
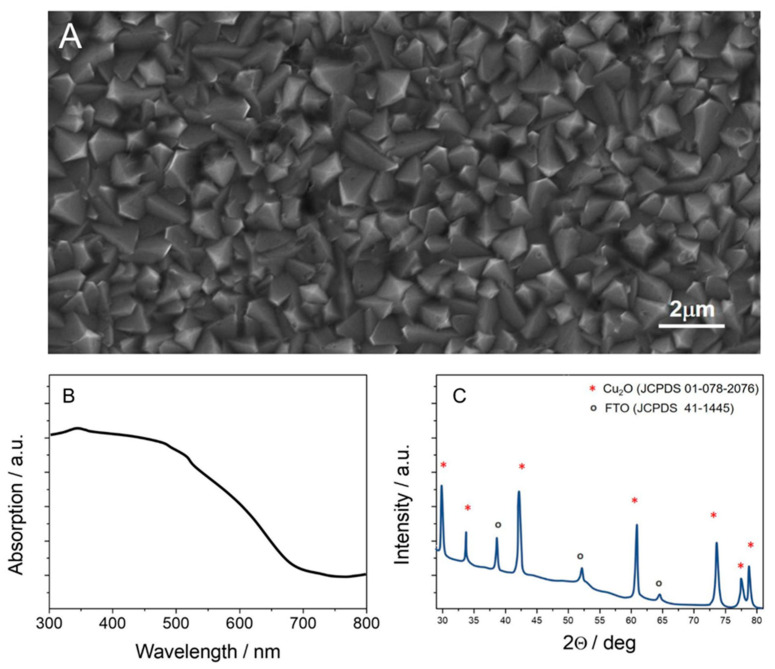
(**A**) SEM picture; (**B**) DRS UV-Vis spectra; and (**C**) XRD pattern of Cu_2_O sample as cast.

The interaction between electrolyte ions and the surface of the working electrode is the most crucial point for achieving the efficient photocatalytic processes occurring on it. The ions present in the electrolyte might affect the processes occurring at the electrode/electrolyte interface mainly through the following fundamental interactions: (1) in the double layer they screen the electric field generated at the working electrode, (2) they influence the transport of the substrates/species involved in the reaction, and (3) they can interact with the electrode surface in a specific way, e.g., become adsorbed on it. Although one can easily find in the literature an investigation of either cation or anion effects on CO_2_RR, there are no publications discussing the combined, simultaneous effect of both cations and anions. Therefore, it is hard to understand the complex processes occurring in the double diffusion layer, since the electrical double layer is composed of both anions and cations. Keeping this in mind, we carried out experiments in the electrolyte consisting of both, differing in size cations and anions with various buffering abilities, such as sulphates, carbonates, and borates, as well. As a working photocathode, a model Cu_2_O film has been chosen. The morphology of such film is shown in Figure 1A.

The morphology of all samples exhibited the typical crystals of copper(I) oxide, approx. 0.7 µm in size. The grain boundaries were very well defined, and the apparent porosity allowed for penetration of the electrolyte within the structure. The thickness of the film was approximately 2 µm (with a deviation of 7% error, determined from the ratio between the deposition charge and the surface area, on which the sample had been deposited). Furthermore, the light absorption properties are important for the photocatalytic performance of the working system.

Thus, a series of electrodes have been investigated with regard to their range of solar energy conversion rates. According to the UV-Vis diffuse reflectance spectra (Figure 1B), all electrodes showed strong absorption in the visible range of the solar spectrum and above 650 nm; therefore, they could be used for CO_2_RR. The band gaps E_g_ of Cu_2_O samples were estimated from the Kubelka–Munk formula and were in the range of 1.9–2.0 eV, depending on their thickness. Compositional analysis of the Cu_2_O samples by XRD (Figure 1C) did not show any other phases. The electrodes exhibited high texture and polycrystallinity (JCPDS 01-078-2076).

### 2.2. Photoelectrochemical Measurements

**Figure 2:**
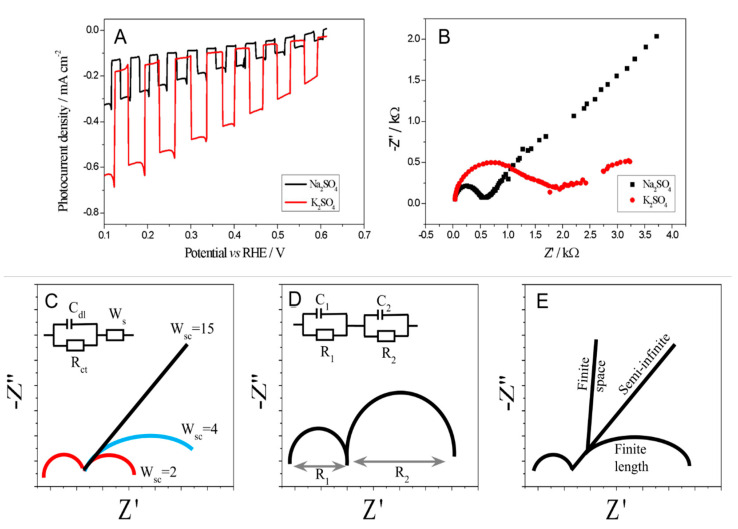
T (**A**) Comparison of photoelectrochemical activities of Cu_2_O photocathode in presence of two different cations (Na^+^ and K^+^); (**B**) Nyquist (imaginary vs. real component of impedance) plots for Cu_2_O photocathode in presence of Na^+^, K^+^ cations under irradiation and imposed potential of 0 V (vs. RHE). All measurements were recorded in either 0.5 M Na_2_SO_4_ (black line) or 0.5 M K_2_SO_4_ (red line) the CO2-saturated aqueous solution. The impedance measurements were performed in the 500 mHz − 1 MHz frequency range with an AC amplitude of 4 mV. Examples of the electrochemical cells operating in: (**C**) the semi-infinite diffusion with Warburg constant in the equivalent circuit and (**D**) the finite length diffusion regime. (**E**) Example of three different areas, where any of the following limiting mechanisms (finite space, semi-infinite, length finite) dominate.

The J-V curves were recorded under the illumination of a 1.5 AM 100 mW × cm^−2^ solar simulator with the use of a model unmodified semiconductor (Cu_2_O) and a clean-surface system with an electrolyte of a specific composition. Such a simple photoelectrochemical system can be approximated by a very simple equivalent circuit consisting of a capacitor, resistor, and a Warburg element, considering diffusion processes. The Nyquist plot of such a simple circuit is shown in Figure 2C. It is important to notice that, for increasing Warburg constants shown in Figure 2C, the second semicircle has an increasing radius, which represents a change in a diffusion mechanism from controlled by linear to semi-infinite diffusion (Figure 2E). Thus, for very small Warburg constants, the system is controlled by linear diffusion and then can be approximated by an equivalent circuit consisting of two resistor-condenser sets (Figure 2D), which represents a typical electrochemical membrane system.

In this regard, we can consider the “rigid layer”, formed upon cation–anion interaction in the proximity of the surface, as a membrane. In consequence, the diffusion layer becomes also more defined. As discussed theoretically by B. Pan [30], the understanding of the role of cations in the formation of the electrical double layer might enable steering the CO_2_RR towards a desirable direction. The CO_2_RR performance in the function of the size of the alkali cations K^+^, Na^+^ (Na_2_SO_4_, K_2_SO_4_) was investigated by comparing the photocurrent and the EIS spectra, as shown in Figure 2A.

In this scenario, under illumination, the sulphates did not show any buffering or complexing properties; therefore, one can conclude that they did not adsorb specifically on the electrode surface. In the presence of K^+^ cation, the photocurrent is much higher, up to two times higher than in the presence of a Na^+^ cation, which is in agreement with the current state of the art. This is also consistent with the recent theoretical considerations of Pan et al. [30], who speculated a significant interaction of large ions (K^+^) with the electrode surface through an uncompensated charge in the cation hydration shell. However, in our case, the observed relationship is much more complex than it was speculated in the article [30]. The K^+^ cations layer resembles a rigid membrane layer at the electrode surface, preventing free diffusion of the intermediate species, protons, etc. However, although this effect largely contributes to a current build-up, the selectivity towards the production of hydrogen instead of CO_2_RR products is much higher, which is not the case when Na^+^ cations are present instead (Figure S1). Nguyen et al. [31] suggested that an improvement in photocurrent occurs by increasing the selectivity towards reduction products and decreasing the diffusion of the intermediate products; however, apart from that, we attribute the observed differences also to the change in the diffusion mechanism from semi-infinite for Na^+^ to linear for K^+^, which is illustrated by the decreasing W constant. It is also important to notice that the charge transfer resistance (R_ct_) for a system with Na^+^ cations has a lower value than for K^+^, which is routinely taken as a good promise for better current performance. At the same time, the capacitance of the electrical double (C_dl_) layer formed in the presence of K^+^ cations increased. Taking into account all the above-mentioned parameters: W, C_dl_, R_ct_, it seems that the photocurrent performance is mostly determined by the W constant.

**Figure 3:**
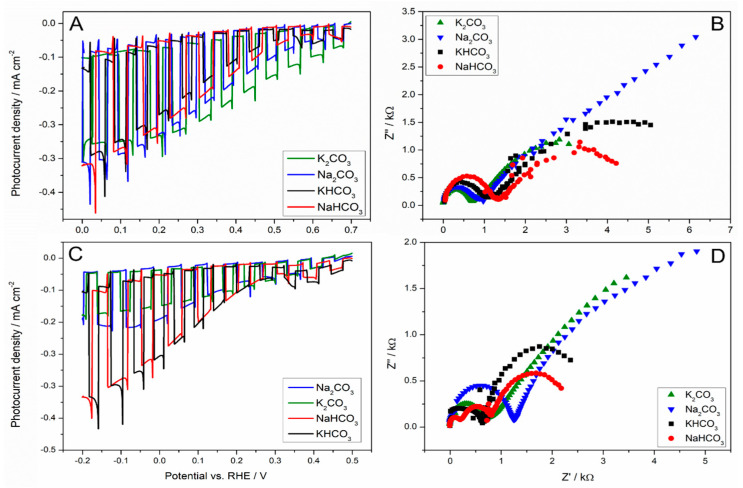
(**A**,**C**) Comparison of PEC activities of Cu_2_O photocathode in the presence of different cations; (**B**,**D**) Nyquist (imaginary vs. real component of impedance) plots of Cu_2_O photocathode recorded in the CO_2_-saturated aqueous solution containing various cations under irradiation and imposed potential of 0 V (vs. RHE). The impedance measurements were performed in the 500 mHz − 1 MHz frequency range with an AC amplitude of 4 mV. All measurements were recorded in the CO_2_-saturated aqueous solution in the presence of one of four different cations: K_2_CO_3_ (green line), Na_2_CO_3_ (blue line), KHCO_3_ (black line), or NaHCO_3_ (red line), at two different concentrations: 0.1 M (**A**,**B**) and 0.5 M (**C**,**D**).

Subsequently, the composition of the electrolyte has been changed to contain carbonates and bicarbonates, which have already been employed in CO_2_RR, either as a carbon source [32] or selectivity modulator, since it has been observed that the yield towards hydrogen production has decreased [29,32]. The literature provides the following mechanisms, by which HCO_3_^−^ influences the CO_2_RR: (i) it might act as a pH buffer [33]; (ii) as a proton donor, it contributes to an increase in an effective CO_2_ concentration in the solution through a rapid equilibrium established between the two species [34]. Considering the two above-mentioned mechanisms, the measurement has been performed in a 0.1 M and 0.5 M solution of Na_2_CO_3_, K_2_CO_3_, KHCO_3_, and NaHCO_3_, saturated with CO_2_. The changes in the photocurrent performance upon the effect of different cations in conjunction with carbonates are shown in Figure 3.

For low concentrations of carbonates and bicarbonates (0.1 M) (Figure 3A), comparable photocurrent values were recorded. However, subtle differences were observed in the electrochemical impedance spectroscopy (EIS) spectra (Figure 3B). An unrestricted diffusion relationship was observed for the sodium cation in the form of Na_2_CO_3_, whereas diffusion was restricted for both bicarbonates and K_2_CO_3_. Consistent with the data (Table 1) from pH measurements before and after CO_2_ saturation, these compounds exhibit weak buffering properties. The carbonate solution had a significantly higher pH after dissolution, while CO_2_ saturation caused a decrease in the pH of the solution. In the case of bicarbonates, the pH change was much smaller upon CO_2_ dissolution.

Thus, if we have cations differing in the size of the hydration layer, then the diffusion becomes linearly controlled in the presence of cations with larger hydrated radii, due to the formation of the rigid surface limiting the diffusion. However, if we consider a system in which we keep the same cation, but we change an anion, then nothing changes if the hydration layer of the cation at OHP is filled, as observed in the case of the Na^+^ cation. In this regard, the CO_2_RR might be only affected by the change in the electric field within the Stern layer (the layer between the surface and OHP). However, if the hydration layer of a cation is smaller, the cations can stabilise CO_2_ and the reaction intermediates through the field–dipole interaction (formation of a so-called ridged surface) [30]. In the presence of K^+^, the interfacial electric field substantially strengthens the adsorption of CO_2_^•^ and other intermediates. Smaller hydration shells can generate stronger electric fields, thereby significantly enhancing partial current densities and Faradaic efficiencies for CO_2_RR [35]. However, as the concentration of salts in the electrolyte was increased (0.5 M), a different cyclic voltammetry (CV) characteristic was observed (Figure 3C). Photocurrent increases as a function of the presence of cations and anions as well, in the following order: KHCO_3_~NaHCO_3_ ˃ Na_2_CO_3_~K_2_CO_3_ (Figure 3C). The correlation of photocurrent with the EIS spectra (Figure 3D), allowed us to notice that only for Na_2_CO_3_ and K_2_CO_3_ the diffusion is semi-finite, while for NaHCO_3_, KHCO_3_ it is controlled by a linear approximation. A reasonable interpretation is that the presence of HCO_3_^−^ anions determines the diffusion, which does not depend on the cation nature anymore. In this scenario, they act as a proton provider; therefore, an overpotential towards the production of hydrogen occurs. This effect is even more pronounced with the increasing concentration of HCO_3_^−^. The simplest explanation for the observed relationships is the formation of hydrogen bonds in the solution containing bicarbonates, which are obviously formed between the hydrogen atom and a lone pair on the oxygen atom in HCO_3_^−^ groups. The layer formed with hydrogen bonds becomes more rigid. Another issue is a charge delocalization in the HCO_3_^−^ structure (in comparison to CO_3_^2−^), which affects the strength of the field dipole interaction.

Boric acid, with its buffering properties and ability to modify surfaces during electrochemical processes, is an electrolyte widely used in electrochemistry. Boric acid can significantly affect PEC CO_2_RR in several aspects since it: (1) increases the amount of CO_2_ dissolved in the electrolyte; (2) possesses buffering properties; (3) inhibits hydrogen evolution. According to Ghosh et al. [36], the step that determines the rate of dissolution of carbon dioxide in aqueous solutions is the following reaction: CO_2_ + OH^−^ → HCO_3_^−^. Boric acid then acts as a catalyst accelerating the above process significantly. The catalysis reaction follows the scheme: B(OH)_3_ + OH^−^ → B(OH)_4_, B(OH)_4_^−^ + CO_2_ → B(OH)_3_ + HCO_3_^−^. Moreover, the reaction product HCO_3_^−^ can be adsorbed on the electrode surface and then starts to act as an intermediate product in CO_2_ reduction [37]. Also, due to the presence of H^+^ ions from H_3_BO_3_ (lowering the pH), the solubility of CO_2_ increases [38]. Furthermore, boric acid possesses buffering properties, which manifest themselves in different ways, depending on the distance from the electrode. As a weak acid, boric acid helps decrease the pH of the solution, which, in turn, increases the amount of H^+^ ions and is related to their presence in processes (proton transfer to CO_2_RR). Given that during the photoreduction process, the pH increases with the overpotential, boric acid can limit the leakage of protons required for CO_2_RR by both lowering the pH of the entire solution and acting as a buffer near the electrode itself.

Inhibition of hydrogen production increases the straightforward selectivity towards CO_2_RR. Boric acid influences ion activity. An explanation given by Wu [39] is that boric acid decreases the average coefficient of ionic activity in solution, causing at the same time an increase in the overvoltage of water reduction. An additional effect is that boric acid adsorbs on the electrode surface and, thereby, effectively reduces the number of active sites on the surface. In consequence, the water reduction reaction (much less demanding than CO_2_RR from a thermodynamic point of view) is inhibited.

**Figure 4:**
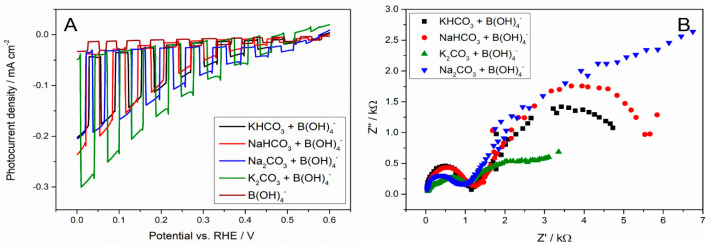
(**A**) Comparison of photoelectrochemical activities of Cu_2_O photocathode in various electrolytes; (**B**) Nyquist (imaginary vs. real component of impedance) plots of Cu_2_O photocathode recorded in various electrolytes under irradiation and at imposed potential of 0 V (vs. RHE). The impedance measurements were conducted in the 500 mHz − 1 MHz frequency range with an AC amplitude of 4 mV. All measurements were recorded in the CO_2_-saturated aqueous solution containing one of the following electrolytes: KHCO_3_/H_3_BO_3_ (black line), NaHCO_3_/H_3_BO_3_ (red line), Na_2_CO_3_/H_3_BO_3_ (blue line), Na_2_CO_3_/H_3_BO_3_ (green line), and boric acid (brown line) at 0.5 M concentration of carbonates, and 3% boric acid.

Figure 4 clearly shows the role of borates in the electric double-layer build-up. In the presence of 3% of borates, in addition to carbonates, the influence of cations is overlapped by properties resulting from the presence of borate anion (among other things, it blocks the surface, inhibits water reduction, has the tetrahedral structure, and the negative charge is strongly shifted to the oxygen). Therefore, the effect of cations on the photocurrent values is almost identical and the system is controlled by linear diffusion, while for boric acid, the current towards CO_2_RR is negligible (brown curve in Figure 4). However, by analysing the EIS spectra, we can clearly see the origin of such a change in the reaction mechanism. Boric acid annihilates the effect of cations and anions and exclusively determines the reaction mechanism due to its comprehensive properties mentioned above.

**Figure 5:**
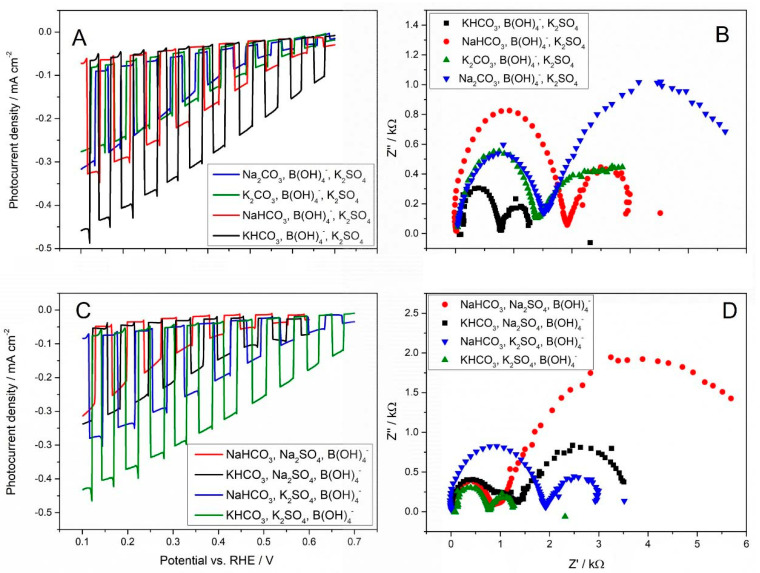
(**A**) Comparison of photoelectrochemical activities of Cu_2_O photocathode in various electrolytes; (**B**) Nyquist (imaginary vs. real component of impedance) plots of Cu_2_O photocathode in various electrolytes recorded under irradiation and at the imposed potential of 0 V (vs. RHE). The impedance measurements were performed in the 500 mHz − 1 MHz frequency range with an AC amplitude of 4 mV. All measurements were recorded in one of the following electrolytes: KHCO_3_/H_3_BO_3_/K_2_SO_4_ (black line), NaHCO_3_/H_3_BO_3_/K_2_SO_4_ (red line), K_2_CO_3_/H_3_BO_3_/K_2_SO_4_ (green line) and Na_2_CO_3_/H_3_BO_3_/K_2_SO_4_ (blue line) in the CO_2_-saturated aqueous solution (0.5 M carbonates and 3% boric acid, 0.5 M Na_2_SO_4_ or 0.5 M K_2_SO_4_). (**C**) Comparison of photoelectrochemical activities of Cu_2_O photocathode in various electrolytes; (**D**) Nyquist (imaginary vs. real component of impedance) plots of Cu_2_O photocathode in various electrolytes recorded under irradiation and at the imposed potential of 0 V (vs. RHE). The impedance measurements were performed in the 500 mHz − 1 MHz frequency range with an AC amplitude of 4 mV. All measurements were recorded in one of the following electrolytes: KHCO_3_/H_3_BO_3_/Na_2_SO_4_ (black line), NaHCO_3_/H_3_BO_3_/Na_2_SO_4_ (red line), KHCO_3_/H_3_BO_3_/K_2_SO_4_ (green line) and NaHCO_3_/H_3_BO_3_/K_2_SO_4_ (blue line) in the CO_2_-saturated aqueous solution (0.5 M carbonates and 3% boric acid, 0.5 M Na_2_SO_4_ or 0.5 M K_2_SO_4_).

Boric acid is a low conductivity electrolyte; however, the significant capacitance value suggests the formation of a double layer with a potentially high charge load (the number of cations and anions). This appears upon the addition of the strong ionic conductor, namely Na_2_SO_4_ (Figure 5A,B) or K_2_SO_4_ (Figure 5C,D). In this scenario, we have carbonates, borates, and sulphate ions in the solution. The presence of sulphate ions helps again to diversify the apparent photocurrent as a function of cation type.

Interestingly, for NaHCO_3_ and KHCO_3_, where we could not observe any differences in the presence of boric acid (Figure 4B), there is again a difference observed in the presence of additional sodium sulphate (Figure 5B), although the capacitance of both systems is almost the same. Therefore, the sodium sulphate presence must affect the properties of the electric double layer in such a way, that the rigid layer is formed. The latter action is favourably affected by the cation size and the field–dipole interaction due to the smaller hydration layer.

Figure 5A shows a better photoelectrode performance in an electrolyte containing potassium bicarbonate, boric acid, and sodium sulphate, as confirmed by the parameters read from the EIS spectrum (Figure 5B). The systems containing Na_2_CO_3_ and B(OH)_4_^−^ behave very similarly to the systems containing NaHCO_3_ and B(OH)_4_^−^ until about 3 kΩ. After that value, the behaviour of both systems is differentiated by the influence of Na_2_SO_4_, where the electrolyte containing Na_2_CO_3_/B(OH)_4_^−^/Na_2_SO_4_ exhibits semi-infinite diffusion and electrolyte with NaHCO_3_/B(OH)_4_^−^/Na_2_SO_4_ shows the linear diffusion limited by the rigid layer with the hydrogen bonds formed between bicarbonate ions. However, the effect of NaHCO_3_, and the buffering effect of B(OH)_4_^−^ are not very noticeable in the presence of Na_2_SO_4_. Evidently, the rigid layer is observed only for the system containing K^+^ ions, and this effect is even more visible in the presence of KHCO_3_ due to the additional effect of hydrogen bond formation by bicarbonate ions. In the case of electrolytes containing KHCO_3_ or K_2_CO_3_, in addition to B(OH)_4_^−^ and Na_2_SO_4,_ a rigid layer is evidently formed that blocks the diffusion of ions. A large value of capacitance of the system containing KHCO_3_ can be interpreted as the inertia of the double layer and can be associated with the existing hydrogen bonds. A similar effect is observed for the KHCO_3_/B(OH)_4_^−^/K_2_SO_4_ as represented by the shape and capacitance value of the black curve in Figure 5D.

It is worth mentioning that the formation of a rigid layer is observed for all the systems presented in Figure 5D. The situation though, is very complex here, and it is very difficult to interpret all of the nuances observed here. For example, the size of the second semi-circle observed for the system Na_2_CO_3_/H_3_BO_3_/Na_2_SO_4_ (blue line) could be sometimes interpreted as a selective adsorption of the substances on the electrode surface. Both systems Na_2_CO_3_/H_3_BO_3_/Na_2_SO_4_ (blue line) and NaHCO_3_/H_3_BO_3_/Na_2_SO_4_ (red line) present relatively small sizes of the second semi-circle, which could be caused by the specific interactions between Na^+^ ions and sulphate ions.

In general, the presence of K_2_SO_4_ is responsible for the formation of a rigid layer and the linear diffusion causing restricted access of ions to the electrode surface. This effect is very similar for the electrolytes containing K_2_CO_3_ (blue line), NaHCO_3_ (red line), and KHCO_3_ (black line), in addition to B(OH)_4_^−^ and K_2_SO_4_. The best photoelectrode performance, though however, is observed for the system containing potassium bicarbonate, boric acid, and potassium sulphate, which is supported by photocurrent values (Figure 5C, black line) as well as the EIS spectrum shape and parameters (Figure 5D, black line). This is in good agreement with the considerations presented above, considering the size of the hydrated potassium ion radius and the size of the tetrahedral sulphate ions.

Table 1 contains the conductivity and pH data recorded in selected electrolytes after saturation with argon or CO_2_, where the electrolytes that exhibited the lowest conductivity values are highlighted in red and those with the highest conductivity values are highlighted in green. As expected, electrolytes with low salt concentrations and those containing boric acid have low conductivity. However, it is interesting to note that Na_2_SO_4_ also has a low conductivity. The difference in mobility of Na^+^ and K^+^ cations clearly supports the observed conductivity values (mobility for Na^+^ 5.19 and for K^+^ is 7.62 (10^−8^ m^2^s^−1^V^−1^) [40]). The conductivity of electrolytes containing K^+^ ions is significantly higher than that of those containing Na^+^ ions. It is also important to mention that the saturation of the solution with CO_2_ has no effect on the electrolyte conductivity.

The second important parameter that has a more profound effect on the properties of the double layer is the pH and buffering properties of the electrolyte. For sulphates and carbonates, the pH change is much more noticeable. Since electrolytes containing boric acid and carbonate ions have buffering properties, the pH change in these systems is much smaller after the saturation with CO_2_. The CO_2_ reduction mechanism is strongly dependent on the concentration of H^+^ [41]. Firstly, pH value determines CO_2_ solubility, as well as the relative concentrations of CO_3_^2−^, HCO_3_^−^, and molecular CO_2_, generating different adsorption pathways. Secondly, the HER hydrogen electroreduction reaction is dominating at low pH values [17]. On the other hand, a high pH environment causes limited availability of protons and the formation of hydrocarbons is significantly restricted [42]. H^+^ ion concentration plays a critical role in determining the preferred reaction mechanism/pathway and, subsequently, the final products [41]. It is crucial then to control the local pH value at the electrode surface. Observed “rigid layer” formation for various electrolytes and the modification of the diffusion mechanism of substrates/products to the electrode surface are important factors that could allow a greater selectivity of the CO_2_ reduction reaction. The last element, which is certainly an important one in relation to pH, is the possibility of hydrogen bonding formation. In the presence of bicarbonate ions and boric acid, water clusters are much more strongly bound by hydrogen bonds, as can be clearly seen in the EIS spectra. Their presence affects the diffusion process, as well as the adsorption on the electrode surface.

**Figure 6:**
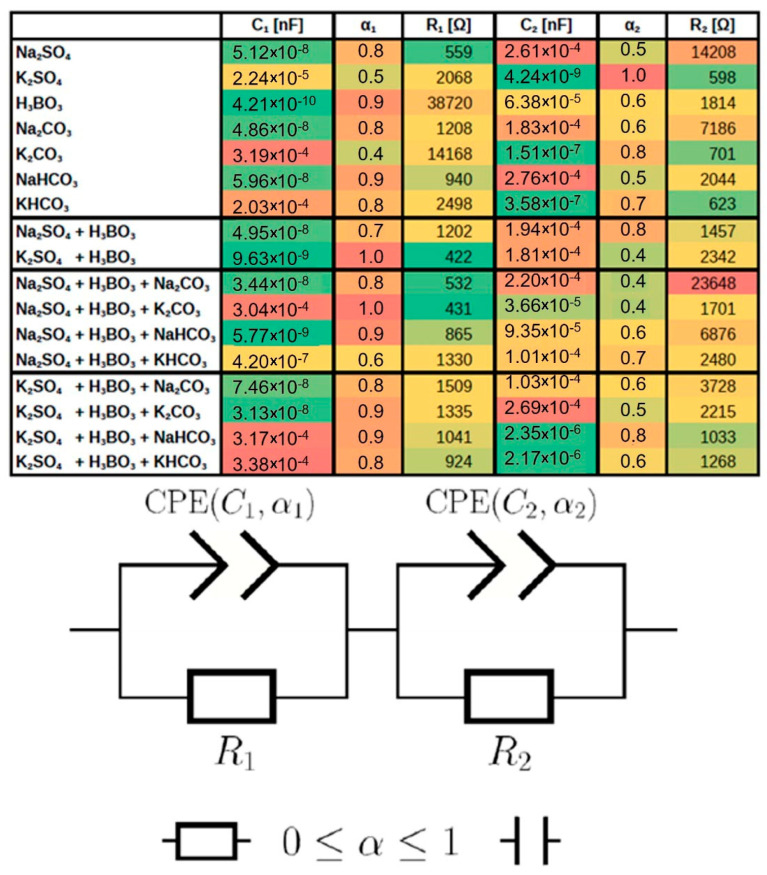
Equivalent circuit parameters for all electrolytes: green colour for lowest values, red colour for highest values. Equivalent circuit used for calculations is shown below. Coefficient α can range from 0 (where CPE acts as resistance) to 1, and CPE was capacitance [43].

Figure 6 shows a summary of the fit of all surrogate systems and the parameters used to fit them. Green has been used for the smallest values and red for the largest for data clarity. The proposed equivalent circuit, consisting of two identical circuits, allows for the matching of all tested electrolytes. The constant phase element (CPE) used, whose parameter a can be in the range from 0 (ideal resistor) to 1 (ideal capacitor), allows for the influence of the electrolyte on the double layer to be analysed [43].

For the first circuit, electrolytes with a K^+^ cation have the lowest a-value, while for the second circuit, we observe the opposite situation—electrolytes with Na^+^ tend to have a lower a-value. Our theory is that the first circuit is identified with a rigid layer near the electrode where the small K cation has the greatest influence, while the second circuit is identified as a diffusive layer. Where the Na cation and its lower mobility are dominant, which is also indeed seen in the R_2_ values. However, according to Meenesh’s work [25], the effect of the cations alone should be linked significantly to the anions. Therefore, systems with buffers (HCO_3_, H_3_BO_3_) show the highest photocurrents.

## 3. Materials and Methods

### 3.1. Chemicals

All chemicals received and used in photoelectrodes’ synthesis as precursors, solvents, and substrates were of an analytical grade. Copper (II) sulphate pentahydrate (CuSO_4_ × 5H_2_O, 98%, Sigma-Aldrich, St. Louis, MO, USA), lactic acid (CH_3_CHOHCO_2_H, MW: 90.08 g/mol, Sigma-Aldrich), sodium hydroxide (NaOH, 98.8%, POCH BASIC), Avantor Performance Materials Poland S.A., Gliwice, Poland), Nafion perfluorinated resin solution (5_wt_ 5% in a mixture of lower aliphatic alcohols and water, containing 15–20% water, Sigma-Aldrich) were used as the precursors for the working electrode. Potassium carbonate (ACS reagent 99%, Sigma-Aldrich), potassium hydrogen carbonate (ACS reagent 99%, Sigma-Aldrich), sodium carbonate (ACS reagent 99%, Sigma-Aldrich), sodium hydrogen carbonate (ACS reagent 99%, Sigma-Aldrich), lithium carbonate (ACS reagent 99%, Sigma-Aldrich), cesium hydrogen carbonate (ACS reagent 99%, Sigma-Aldrich), ammonium hydrogen carbonate (ACS reagent 99%, Sigma-Aldrich), and orthoboric acid (H_3_BO_3_, ACS reagent 99%, Sigma-Aldrich) saturated either with argon (Ar, 99.999%, Kamino, San Diego, CA, USA) or carbon dioxide (CO_2_, 99.999%, Messer, Bad Soden, Germany) were used as a supporting electrolyte subjected to the reduction process. Methanol (CH_3_COHCH_3_OH, 99.8%, POCH BASIC, Gliwice, Poland) Avantor Performance Materials Poland S.A.) and carbon monoxide (CO, 99.999%, Messer) were used as reference gases for the detection of the reduction products by the gas chromatography equipped with the BID and mass spectroscopy detectors (GC–MS). The electrolyte solutions were prepared with deionized water of a resistivity of about 18 Ω cm.

### 3.2. Materials

The electrodeposition of the copper (I) oxide has been described in detail elsewhere [9,44]. Briefly, the preparation of a working electrode was performed with a standard three-electrode system on an FTO substrate (1.5 cm × 3 cm). The Cu_2_O layer on the FTO substrate was electrodeposited from lactate stabilised copper (II) sulphate. Pt wire was employed as the counter electrode and Mercury Sulphate Electrode (MSE, Hg/Hg_2_SO_4_) served as the reference electrode. A clear precursor solution was prepared following the dissolution of 1.92 g of Cu_2_SO_4_·5H_2_O and 6.71 mL of lactic acid in 30 mL of deionized water. The precursor solution was then adjusted to pH 9 by the controlled addition of sodium hydroxide crystals. Subsequently, the copper (I) oxide film was electrodeposited onto the FTO glass substrate (FTO, resistivity ≈ 8 Ω/cm^2^, Sigma Aldrich) from the precursor solution at 0.64 V vs. MSE. The temperature of the reaction bath was kept at 60 °C for 1800 s during the whole cathodic electrodeposition run. The CH Instruments electrochemical workstation was used to control, impose, and maintain a potential during the chronoamperometric Cu_2_O film deposition at the conductive support. Thereafter, the resulting film of Cu_2_O was calcined in air at 150 °C for 30 min.

### 3.3. Characterisation Methods

#### 3.3.1. Structural, Optical

The structural characterisation of the prepared films was performed through the X-ray diffraction (XRD) method on the X’Pert PRO MPD (London, UK) equipped with a Si [Li] solid-state detector. The crystal structure and phase identification were made by XRD using Co-K_α_ radiation and a secondary monochromator in the range of 2θ from 10 to 80. The morphologies of the produced films were analysed by means of scanning electron microscopy (SEM) using a Zeiss (Oberkochen, Germany) microscope equipped with two detectors: SE2 and InLens. Diffuse reflectance spectra of the produced samples were measured by using a Jacso V650 UV–Vis (Tokyo, Japan) spectrometer equipped with a 16 mm integrating sphere.

#### 3.3.2. Electrochemical

Electrochemical impedance spectroscopy (EIS) data were acquired using an AC perturbation of 4 mV (in amplitude) and within 500 mHz − 1 MHz frequency range, under illumination and at the selected DC potentiostatic conditions (0 V vs. RHE). Nyquist plots (correlation of imaginary vs. real components of impedance, Z_Im_ vs. Z_Re_) are presented as received experimentally.

The conductivity of a given electrolyte was measured with Elmetron (Zabrze, Poland) CC-401 conductivity metre (averaged across 3 electrodes: k = 9.660 cm^−1^, k = 0.481 cm^−1^, k = 1.028 cm^−1^) and pH was controlled by the Elmetron CPO-401 pH metre.

All electrochemical measurements were performed with the use of a CHI electrochemical workstation (CH Instruments model 660 E).

#### 3.3.3. Photoelectrochemical

The photocurrent-potential profiles for CO_2_ reduction processes were recorded in a two-compartment photoelectrochemical cell by scanning the potential from −0.5 V to −1.1 V vs. MSE (at 10 mV s^−1^) under simulated solar irradiation. The solar simulator of AM 1.5 G irradiation was provided by LOT Oriel (Pfungstadt, Germany) and equipped with a 150 W Xe lamp. The light spectrum was fitted with an AM 1.5 Global 2 × 2-inch filter (model AM 1.5G, Instytut Fotonowy). Light intensity was adjusted to 100 mW × cm^−2^ with a calibrated reference cell (Portable Radiometer, International Light Technologies, Peabody, MA, USA) model ILT 1400 with a SEL623 Thermopile detector and NIST Traceable Calibration. The current versus potential (J-E) plots for Cu_2_O photocathodes were recorded in a Teflon cell equipped with a quartz window. The exposed Cu_2_O electrode surface area was 0.28 cm^2^.

The supporting electrolyte (used for the reduction experiments) was saturated with argon and then with CO_2_ for 30 min. To overcome the problems associated with low stability and photocorrosion of the Cu_2_O, many synthesis conditions have been tested to optimise the electrode properties. Nevertheless, each photoelectrochemical measurement was made with the use of a new Cu_2_O electrode in order to obtain reproducible and reliable results. The thickness of each electrode was determined from the ratio between the deposition charge and the electrode surface area. The discrepancies in their thicknesses were not higher than 7%.

#### 3.3.4. Product Detection

The product analysis was carried out using a gas chromatograph GCMS-QP2020 with a BID detector (Shimadzu, Kyoto, Japan). Ar (99.9999%) was used as the carrier gas. Measurements were taken for both gaseous products and the electrolyte, with 2 µL of the sample aliquot taken directly each time. The reaction products were detected over a 1-h-long photocatalysis run due to the instability of the bare Cu_2_O electrode after that time.

## 4. Conclusions

Various interactions involving the role of cations in the formation of a so-called “rigid surface” near the electrode, as well as cation–anion interactions and anion buffering properties, might influence the electric double layer’s build-up. All these interactions were investigated towards achieving a more effective and selective CO_2_RR. We found out that cations with a smaller ionic radius and a larger hydration layer showed lower performance in CO_2_ conversion due to a higher W constant related to a diffusion model. In contrast, a significantly higher photocurrent was observed in the presence of cations with a larger ionic radius and a smaller hydration sphere. This effect has been explained by the formation of the “rigid surface” and a subsequent change in the diffusion mechanism from unlimited (for small cations) to linearly limited (for large cations). The influence of different anions on the CO_2_RR mechanism and its efficiency was analysed in the presence of buffering carbonates, borates, and highly conductive sulphates. We found that anions with buffering properties (B(OH)_4_^−^ and HCO_3_^−^) had a significant effect on the CO_2_ reaction mechanism also through specific adsorption to the electrode surface, acceleration of CO_2_ dissolution, and stabilisation of reaction intermediates, while SO_4_^2−^ and CO_3_^2−^ anions did not significantly affect the CO_2_ photoreduction process.

The CO_2_ reduction process is a complex process, and the influence of the electrolyte is one of the important elements for achieving a high performance, efficient process. Only a comprehensive analysis of the electrolyte, namely the influence of cations and anions on the double layer formation and its composition, might allow us to better understand the whole picture.

## Figures and Tables

**Table 1:** Conductivity and pH data in various electrolyte solutions containing Ar or CO_2_.

pH	Conductivity [mS]	Electrolyte
9.15	37	Na_2_SO_4_, NaHCO_3_, Ar
7.7	40	Na_2_SO_4_, NaHCO_3_, CO_2_
9.02	47	Na_2_SO_4_, KHCO_3_, Ar
7.55	47	Na_2_SO_4_, KHCO_3_, CO_2_
11.44	57	Na_2_SO_4_, Na_2_CO_3_, Ar
8.99	57	Na_2_SO_4_, Na_2_CO_3_, CO_2_
9.03	15	Na_2_SO_4_, Ar
4.39	15	Na_2_SO_4_, CO_2_
8.46	33	Na_2_SO_4_, NaHCO_3_, Ar, H_3_BO_3_
7.45	36	Na_2_SO_4_, NaHCO_3_, CO_2_, H_3_BO_3_
8.52	42	Na_2_SO_4_, KHCO_3_, Ar, H_3_BO_3_
7.48	43	Na_2_SO_4_, KHCO_3_, CO_2_, H_3_BO_3_
9.53	48	Na_2_SO_4_, Na_2_CO_3_, Ar, H_3_BO_3_
7.85	51	Na_2_SO_4_, Na_2_CO_3_, CO_2_, H_3_BO_3_
8.64	14	Na_2_SO_4_, H_3_BO_3_, Ar
7.49	14	Na_2_SO_4_, H_3_BO_3_, CO_2_
11.37	49	Na_2_CO_3_, Ar
7.87	48	Na_2_CO_3_, CO_2_
9.76	41	Na_2_CO_3_, H_3_BO_3_, Ar
8.87	43	Na_2_CO_3_, H_3_BO_3_, CO_2_
9.04	25	NaHCO_3_, Ar
7.67	27	NaHCO_3_, CO_2_
8.99	22	NaHCO_3_, H_3_BO_3_, Ar
7.7	25	NaHCO_3_, H_3_BO_3_, CO_2_
9.28	39	KHCO_3_ 0.5 M, Ar
7.78	40	KHCO_3_ 0.5 M, CO_2_
8.93	33	KHCO_3_, H_3_BO_3_, Ar
7.79	35	KHCO_3_, H_3_BO_3_, CO_2_
4.17	44	K_2_SO_4_, CO_2_
7.41	43	K_2_SO_4_, H_3_BO_3_, CO_2_
8.81	98	K_2_SO_4_, K_2_CO_3_, CO_2_
7.55	83	K_2_SO_4_, KHCO_3_, CO_2_
8.6	95	K_2_SO_4_, NaHCO_3_, CO_2_
7.48	106	K_2_SO_4_, NaHCO_3_, CO_2_
8.62	101	K_2_SO_4_, K_2_CO_3_, H_3_BO_3_, CO_2_
8.54	84	K_2_SO_4_, KHCO_3_, H_3_BO_3_, CO_2_
8.22	97	K_2_SO_4_, Na_2_CO_3_, H_3_BO_3_, CO_2_
8.33	85	K_2_SO_4_, NaHCO_3_, H_3_BO_3_, CO_2_
11.3	64	K_2_CO_3_, Ar
7.6	66	K_2_CO_3_, CO_2_
11.064	17	K_2_CO_3_ 0.1 M, Ar
7.107	15	K_2_CO_3_ 0.1 M, CO_2_
8.292	9	KHCO_3_ 0.1 M, Ar
6.936	8	KHCO_3_ 0.1 M, CO_2_

## Data Availability

The data presented in this study are available on request from the corresponding author.

## Supplementary Materials

The following supporting information can be downloaded at: https://www.mdpi.com/article/10.3390/molecules30040885/s1, Figure S1: O_2_ reduction product distribution over Cu_2_O in 0.5 M K_2_SO_4_ saturated by CO_2_ (on the left side of the graph), Cu_2_O in 0.5 M Na_2_SO_4_ saturated by CO_2_ (in the middle) and a control sample under dark (on the right side of the graph); Figure S2: chronoamperometric: photocurrent density versus time (i-t) curves recorded at the imposed potential of 0.3 V vs. RHE in K_2_SO_4_ (black curve), K_2_SO_4_ + KHCO_3_ (red curve) and K_2_SO_4_ + KHCO_3_ + 3% H_3_BO_3_ (blue curve), K_2_SO_4_ (magenta curve) solutions saturated with CO_2_.
