# Supplementary material for: Influence of Electrolyte Composition on the Semiconductor–Electrolyte Interface (SEI) Built-In for Enhanced Photoelectrochemical (PEC) Processes
Source: Molecules. 2025 Feb 14;30(4):885. doi: 10.3390/molecules30040885 (PMC11858421; doi:10.3390/molecules30040885)
Supplement: Supplementary file 1 [file molecules-30-00885-s001.zip › molecules-3317564-supplementary.pdf]

molecules

MDPI

Influence of electrolyte composition on the semiconductor electrolyte interface (SEI) built-in for enhanced photoelectrochemical (PEC) processes

Bartosz Leks[1], Aleksandra Parzuch[1], Nabila Nawaz[1], Justyna Widera[2], Krzysztof Bienkowski[1]*, Renata Solarska[1]*

[1] Laboratory of Molecular Research for Solar Energy Innovations, Centre of New Technologies; University of Warsaw, Banacha 2c, Warsaw, Poland k.bienkowski@cent.uw.edu.pl, b.leks@cent.uw.edu.pl, a.parzuch@cent.uw.edu.pl, n.nawaz@cent.uw.edu.pl, r.solarska@cent.uw.edu.pl

[2] Department of Chemistry, Adelphi University, 1 South Avenue, Garden City, NY 11530, USA, widera@adelphi.edu

* Correspondence: k.bienkowski@cent.uw.edu.pl, r.solarska@cent.uw.edu.pl, +48690818700.

Supplementary Information

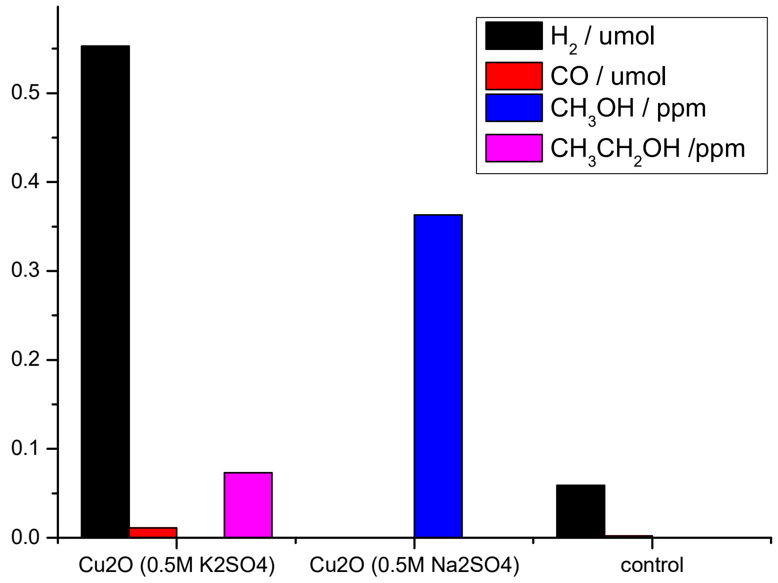

Figure S1. CO_2 reduction product distribution over Cu_2O in 0.5M K_2SO_4 saturated by CO_2 (on the left side of thr graph), Cu_2O in 0.5M Na_2SO_4 saturated by CO_2 (in the middle) and a control sample under dark (on the right side of the graph)

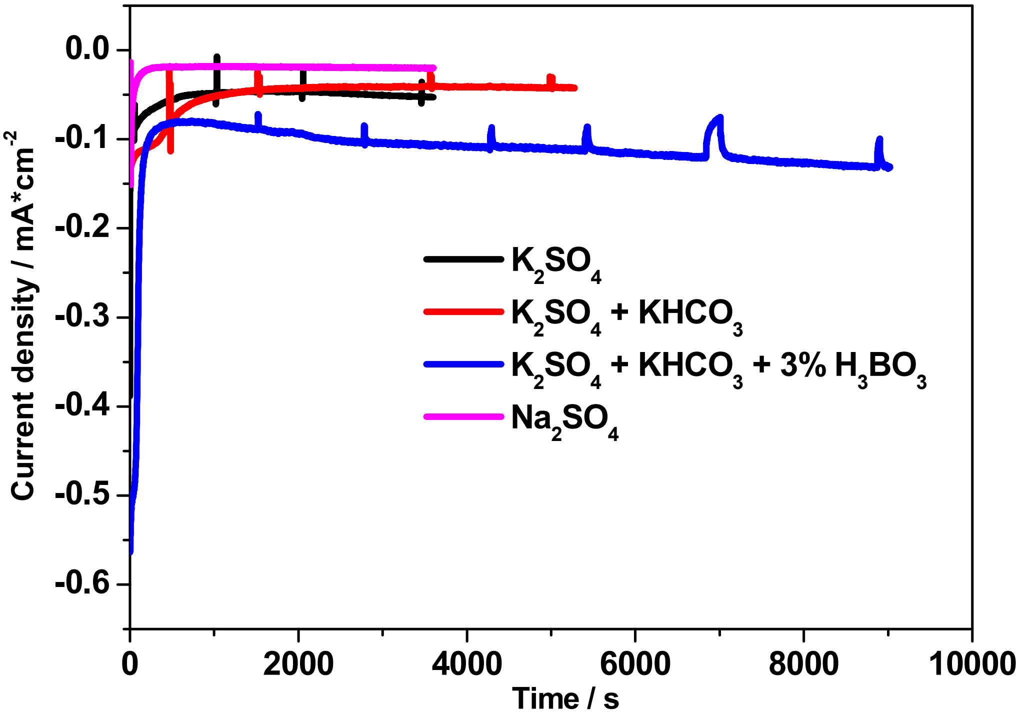

Figure S2. Chronoamperometric: photocurrent density versus time (i-t) curves recorded at the imposed potential of 0.3 V *vs* RHE in K_2SO_4 (black curve), K_2SO_4 + $KHCO_3$ (red curve) and K_2SO_4 + $KHCO_3$ + 3% H_3BO_3 (blue curve), K_2SO_4 (magenta curve) solutions saturated with CO_2
